# Developing a Strategy for COVID-19 Control Among Hard-to-Reach Migrant Communities: The Experience of Roma 2 Local Health Authority

**DOI:** 10.3389/ijph.2023.1605766

**Published:** 2023-11-03

**Authors:** Annalisa Rosso, Alessandro Rinaldi, Daniele Coluzzi, Fabrizio Perrelli, Pier Angela Napoli, Maria Elena Flacco, Lamberto Manzoli, Giuseppe De Angelis

**Affiliations:** ^1^ Migrants Health Unit, Local Health Authority Rome 2, Rome, Italy; ^2^ Department of Environmental and Preventive Sciences, University of Ferrara, Ferrara, Italy; ^3^ Department of Medical and Surgical Sciences, University of Bologna, Bologna, Italy; ^4^ Department of Prevention, Local Health Authority Rome 2, Rome, Italy

**Keywords:** COVID-19, migrant and refugee health, hard-to-reach, COVID-19 vaccination, migrant health

## Abstract

Roma 2 Local Health Authority (ASL) developed a strategy to control the COVID-19 epidemic in Hard-to-reach (HTR) migrant communities, addressing both the containment of clusters in informal settlements and access to COVID-19 vaccination. The strategy was based on a strong collaboration of different services across the ASL and with Non-Governmental Organizations (NGOs). NGOs were involved in the active surveillance, reporting of COVID-19 suspected cases to the ASL and information to the communities. Health interventions (e.g., COVID-19 tests, contact tracing, vaccination) were offered in outreach in HTR communities’ life places. From April 2020 to February 2021, 15 outbreaks were controlled, for a total of over 4,500 persons reached, and 265 COVID-19 cases identified. From July to November 2021, vaccinations were offered in outreach or with dedicated sessions, reaching 1,398 people. This intervention model may lay the foundations for the design of public health strategies, not only aimed at HTR populations.

## Background

The impact of COVID-19 pandemic on health systems has disproportionately affected individuals from disadvantaged socio-economic backgrounds, such as the poor, those living in deprived areas, migrants and ethnic minorities, who were already facing difficulties in finding answers to their own health needs [1]. Migrant populations showed a higher vulnerability to COVID-19 due to their living conditions (often characterized by overcrowding and multigenerational coexistence), their work situation (they often face occupational risks or precarious working conditions), and difficulties in accessing public health messages and services, including vaccination [2]. Such difficulties were more evident in certain subgroups characterized by greater social marginality (e.g., undocumented migrants, Roma communities, people living in informal settlements) [2], which can be included within the wide definition of “hard-to-reach (HTR)” [3].

HTR migrant populations are widely represented in the territory of Roma 2 Local Health Authority (ASL- Azienda Sanitaria Locale). While the exact number of undocumented migrants living in Roma 2 area is unknown, according to data collected by the NGO Medecins Sans Frontier (MSF) in 2018, the territory hosts 21 informal settlements, where approximately 5,000 people live, mostly inside disused buildings and in degraded urban areas, with difficulties in water supply, sanitation and overcrowding [4]. It also hosts a large community of roma people, distributed in five large camps-where approximately 1,800 people live- and several small informal settlements where another 500 people are estimated to be living [5].

Since the beginning of the epidemic, ASL Roma 2 Migrants Health Unit (MHU) identified several barriers to guarantee COVID-19 control in HTR migrant populations, including difficulties in the diagnosis of SARS-Cov-2 infection, conducting contact tracing and surveillance activities, and in accessing COVID-19 vaccination campaign. A multi-component collaborative strategy was thus developed to guarantee COVID-19 control in these population groups, with a focus on the containment of clusters in closed communities and on promoting access to COVID-19 vaccination.

Based on previous collaborations, the MHU identified five NGOs working in the provision of healthcare, information and education on COVID-19 to HTR migrant communities living in ASL Roma 2 territory: MSF Italy, Medecins du Monde MdM-Italy, Medici per i Diritti Umani- MEDU, Intersos and Sanità di Frontiera ONLUS- SDF.

Starting from March 2020, periodic coordination meetings were held by the MHU and its partner organizations with the aim to develop a strategy for the prompt identification of suspected and confirmed COVID-19 cases in HTR communities. With the beginning of the vaccination campaign in January 2021, the focus of the coordination mechanism switched to developing a strategy to promote access to vaccination for HTR communities, mainly undocumented migrants. Roles and responsibilities of the Local Health Unit and partner organizations were defined in a Memorandum of Understanding signed by the legal representatives of both parties.

## Management of SARS-Cov-2 Clusters in HTR Settings

Based on the main barriers to access SARS-Cov-2 control services, a multi-component collaborative strategy was developed, including the following steps:

• Active surveillance and monitoring of target communities: NGOs and ASL Roma 2 mobile unit health staff conducted regular visits to informal settlements to identify symptomatic individuals or contacts/confirmed COVID-19 cases;
• Notification of suspected/confirmed cases to the MHU staff, who conducted site-visits together with NGOs to meet community representatives, conduct preliminary case investigation (with the support of cultural mediators, if needed, and community members) and assess the local epidemiological (number of people/families testing positive, type of contacts across community members), structural (e.g., presence of shared bathrooms/kitchens) and organizational (presence of recognized community leaders) context;
• Prompt development and coordination of control measures, including timely transfer of positive cases to dedicated isolation facilities (COVID hotels), on-site testing of all close contacts/all community members (depending on the local epidemiological context), delivery of information on isolation/quarantine measures;
• Monitoring of quarantined contacts: NGOs and ASL Roma 2 mobile unit staff conducted regular visits to the settlements to monitor the health status of quarantined contacts, informing the MHU of each new symptomatic individual so as to program a test and transfer new cases to isolation facilities as soon as possible.

The strategy envisaged a strong internal collaboration across different services of ASL Roma 2:

• the Hygiene and Public Health Service (SISP- Servizio di Igiene e Sanità Pubblica) defined together with the MHU the most appropriate isolation and quarantine measures, based on the situation assessment;
• a specific nursing team established within the COVID-19 pandemic, called Homecare COVID, was responsible of performing nasopharyngeal swabs on-site;
• relocated administrative staff from the ASL Booking System (CUP- Centro Unico di Prenotazione) were responsible for collecting anagraphic data, labelling swabs and registering SARS-Cov-2 antigenic test results on-site.

The level of control measures adopted to manage each cluster differed depending on the local epidemiological, structural and organizational context. Outbreaks happening in settlements characterized by high levels of internal organization, good communication with the local population, housing units with private bathrooms (or possibility to dedicate some bathrooms for private use to cases) were managed following official recommendations for the general population, with case isolation and quarantine at home for close contacts, and testing of contacts to end quarantine or upon presentation of symptoms (Level 1). In case of weaker coordination mechanisms or unclear transmission patterns within the settlement, testing was offered to all inhabitants of the settlement on a voluntary bases (Level 2). At the beginning of the pandemic, in some cases were a high SARS-Cov-2 transmission rate was immediately detected in the settlements, the control strategy envisaged quarantining of the entire community, either without (where a strong collaboration of community referents was possible, Level 2), or with legal enforcement (Level 1). In these cases, social support was guaranteed (food, medication and other basic needs were provided) along with medical supervision of quarantined people by partner medical organizations. The level implemented in each situation is summarized in Table 1.

**TABLE 1:** Description of SARS-COV-2 clusters by settlement (Roma, Italy March 2020–March 2021).

Settlement	Time	Type of intervention	Estimated N. inhabitants	N. Tested	N. Positive	Test positivity rate (%)
Selam Palace I^a^	March 2020	Level 4	509	509	39	7.7
Roma Camp“La Barbuta”^a^	May 2020	Level 1	586	20	1	5.0
Pecile^a^	June 2020	Level 4	107	107	25	23.4
Caravaggio^a^	September 2020	Level 3	253	251	9	3.6
“La Rustica”^a^	September 2020	Level 2	221	220	66^b^	30.0
Hotel 4 stelle^a^	September 2020	Level 2	399	358	23	6.4
Collatina^a^	September 2020	Level 2	300	237	26	11.0
Tor de Schiavi	October 2020	Level 1	32	32	11	34.4
Sambuci	October 2020	Level 1	200	39	18	46.1
Casale De Merode	October 2020	Level 1	200	26	2	7.7
Tiburtina n. 1064	October 2020	Level 1	200	50	5	10.0
Tiburtina n. 770	October 2020	Level 1	150	31	2	6.5
Selam Palace II	October 2020	Level 1	509	125	9	7.2
Roma Camp “Salone”	November 2020	Level 1	607	36	3	8.3
Roma Camp“Castel Romano”	February 2021	Level 1	400	270	26	9.6
Total			4,673	2,311	265	11.47

^a^ SARS-Cov-2 screening test offered to the total population.

^b^ The total refers to positive cases reported during a 2 months continuous surviellance.

As a result of these actions, from March 2020 to February 2021, 15 epidemic clusters were managed by the ASL Roma 2 MHU in 14 different settlements (two different clusters were registered in the Selam Palace occupation, in March and October 2020), 12 of them in occupied buildings and three in roma camps. Based on estimates obtained from the mapping of settlements and from censuses conducted by Rome Municipality officers in roma camps, the total population reached was of over 4,000 people: nearly half of them were reached by contact tracing activities and tested for SARS-Cov-2, with an overall positivity rate of 11.47% (Table 1).

In May 2021 the MHU transferred the knowledge acquired and the model developed to ASL Roma 2’s SISP, responsible for the overall control of the COVID-19 epidemics in the territory.

## Promotion of COVID-19 Vaccination Among HTR Groups

**FIGURE 1:**
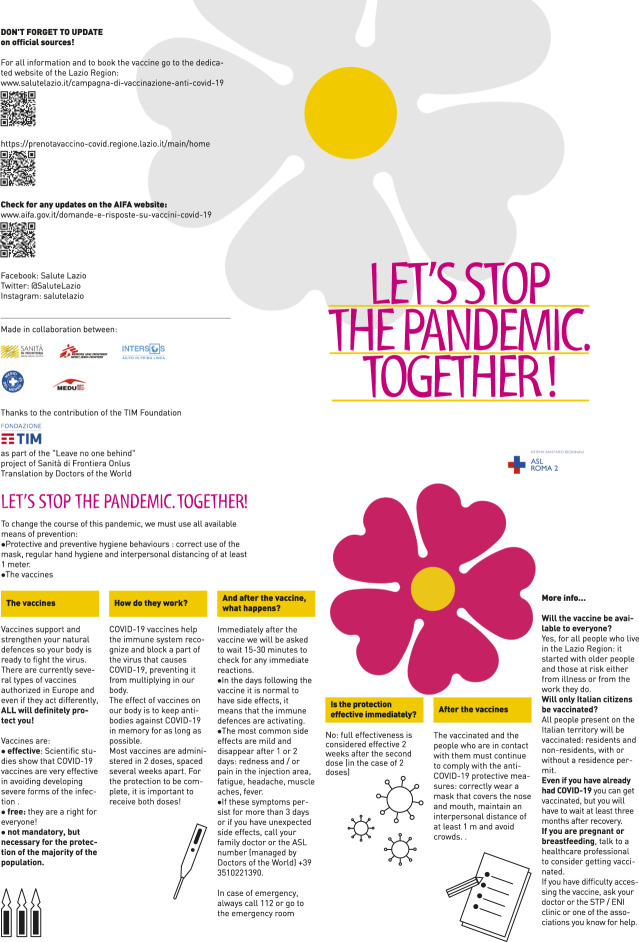
Informative leaflet on COVID-19 vaccination developed by ASL Roma 2 and partner organizations (English version) (Roma, Italy March 2020–March 2021).

NGOs staff surveyed vaccination coverage and barriers to access vaccinations in HTR communities, also including an assessment of knowledge and attitudes on the COVID-19 vaccine. Based on the most frequently asked questions and doubts raised on available vaccines, an informative leaflet was co-designed by the MHU and the partner organization, translated in nine languages other than Italian (English, French, Spanish, Arabic, Amaric, Bengali, Romanian, Somali, Tigrinya) and distributed to different audiences, as previously described [6] (see Figure 1). The graphics used recalled those of the official vaccination campaign, with the primrose symbol. Due to time constraints, it was not possible to test the informative material with local migrant communities before dissemination.

The main barriers identified to access COVID-19 vaccination were administrative issues (mainly lack of documents and enrollment in the National Health System, not allowing to register for the vaccination programme) and a low level of knowledge on the available vaccines. Thus, the strategy to increase vaccination uptake in these population subgroups included the following:

• Information and orientation campaigns conducted by NGOs with HTR communities, through regular visits, production and distribution of an informative leaflet;
• Organization of dedicated vaccination sessions, intended for widespread communities with no access to the ordinary vaccination campaign (e.g., undocumented migrants);
• Outreach vaccination activities for closed communities, such as people living in roma camps.

Connections were established with various community based organizations (e.g., Dhuumcatu, organization of the Bengalese community living in Rome, Nonna Roma, association providing social support to homeless people and marginalized communities), to spread information on the dedicated vaccination sessions’ dates and venues and mode of registration. An online registration system was created with a dedicated link on ASL Roma web-site. During the sessions, the presence of cultural mediators speaking the most represented languages (Bengalese, Spanish and Arabic language) and administrative staff issuing STP Cards (cards issued by the Regional Health Service for non-EU citizens irregularly staying in the territory to access urgent, essential and ongoing treatments) was guaranteed.

In July 2021 nine vaccination sessions were organized dedicated to foreigners without a residence permit, and more generally to people who could not access vaccination through official channels, in the vaccination center located in via La Spezia, Rome. Janssen^®^ vaccination was offered as per the Ministry of Health recommendation as the preferred choice for HTR populations, by virtue of the single-dose schedule [7]. A total of 824 people were vaccinated out of 968 who had registered on ASL Roma 2 website (85.1%). The geographical origin was very varied, also including Italian people who faced administrative barriers in accessing the regular vaccination campaign (e.g., people residing in a different Region). People from Bangladesh and Perù represented nearly half of people vaccinated (34.0% and 13.8%, respectively). The mean age was 35.9 years.

In consideration of the high number of requests, additional sessions were programmed in August in La Nuvola Vaccination Hub, where the regular vaccination campaign was running, in a dedicated area where also administrative staff and cultural mediators were present. In this case, vaccination was offered with Spikevax^®^ (Moderna) in eight sessions. A total of 500 people out of 790 booked (63.3%) were vaccinated with at least one dose, 70.4% of whom (352 people) completed the vaccination schedule in both sessions. From the partial data collected on vaccinated subjects, the most represented nationalities were Bangladesh (representing nearly half the vaccinated), Peruvian and Chinese. The mean age of vaccinated people was 31.9 years.

In addition to the dedicated vaccination sessions, the NGO Sanità di Frontiera carried out information and orientation activities in three roma camps located in ASL Roma 2 territory (via Salone, via dei Gordiani, via Salviati), where an estimated population of 1,000 people is living. During their work they identified people interested in being vaccinated who were not able to access the official campaign due to administrative barriers, and outreach activities were organized to offer COVID-19 vaccine to these groups. From October 2021 to January 2022 a total of 74 people living in roma camps were vaccinated in outreach, including 44 inhabitants of “Gordiani” camp and 30 from “Salone”. Also in these sessions, vaccination was offered with Spikevax^®^. “Salviati” camp was not included in the outreach activities, being the demand from its inhabitants very low.

## Discussion

From April 2020 to February 2021, the multicomponent collaborative strategy developed by ASL Roma 2 Migrants Health Unit and its partners reached approximately 4,500 persons and allowed the prompt identification of 293 COVID-19 cases in HTR contexts, where people were facing barriers in accessing services for SARS-Cov-2 diagnosis and control. A study conducted by the Italian National Institute of Health showed a delay in the diagnosis of infection in foreign citizens compared to Italians due to a delayed use of health services by these populations, leading to a greater risk of hospitalization, but also a greater risk of transmission to the population [8]. Thus, our interventions had both an impact on the improvement of patients’ prognosis and on the interruption of SARS-Cov-2 chain of transmission. Furthermore, during the period July–November 2021, 1,398 people were vaccinated who would have not accessed COVID-19 vaccine otherwise, thanks to the organization of dedicated sessions and outreach activities.

The strategy was based on previous experiences carried out in the context of migrants and HTR communities’ health in Italy, which have been framed under the definition of “Proximity Public Health” [9]. According to this definition, access to prevention and care resources in HTR groups can be promoted through the active provision of healthcare outside clinical settings (outreach), the reorientation of healthcare services with a view to greater permeability and usability (system mediation) and the involvement of the population in empowerment processes. Our interventions included all these elements.

Proximity Public Health strategies also envisage a strong collaboration between public institutions, social organizations and communities in a given territory, as it happened in our intervention model. In these regards, it has to be stressed that our results could be achieved only by virtue of the active involvement of different ASL Roma 2 services (MHU, SISP, Homecare COVID, Vaccinations Unit), and of a strong collaboration with other actors, in particular NGOs.

The presence of an operational unit dedicated to the protection of migrants and vulnerable populations’ health represented an added value in the development of this strategy. ASL Roma 2 could count on an already established network and on previous experiences of Proximity Public Health interventions in the territory. Based on our experience, we would thus recommend setting up of a dedicated unit/group of experienced professionals dedicated to guaranteeing access of migrants and HTR communities within public health services, particularly in territories characterized by a high presence of migrant and socially vulnerable groups.

The European Centre for Disease Prevention and Control (ECDC) recommended EU Member States to promote and develop policies and strategies offering to migrants in precarious social situations the possibility to test, self-isolate, access health and social services, and better engage with contact tracing mechanisms and mass (asymptomatic) testing [2]. It also stressed that the specific needs of migrants who encounter barriers to accessing health services (such as people without residence permits or documents, those living in settlements, refugees and asylum seekers), should be taken into account in national response plans. While the model developed in Rome 2 territory has provided for actions complying with these recommendations, both at the national and regional level in Italy no policy indications were provided to guarantee access of non-regular migrants to COVID-19 prevention, diagnosis and treatment. Similarly, while international public health agencies have stressed the importance of including migrant populations as a priority target in vaccination campaigns [2, 10, 11], the Italian National Strategic Plan for SARS-Cov-2 vaccination did not explicitly mention migrant populations, nor more generally people with social vulnerability, as a priority category for vaccination, including a risk stratification based on co-morbidities and age only. In general, it did not provide any specific indication on the need to ensure access to vaccination for migrants regardless of their legal situation (absence of residence permit/residence, etc.) [12]. Thus, despite our organizational effort, several structural barriers were in place, mainly during the first 2 years of the pandemics, whose overcoming could have guaranteed a much easier access to a large number of people to COVID-19 control measures.

During the development of our strategy, we mainly collected process indicators, aimed at monitoring the actions undertaken to reach, test, isolate and vaccinate the target population. The main limitation of our model lies in the impossibility to provide data on the outcomes and impact of the interventions conducted. This is due both to the type of interventions offered (e.g., CT activities, vaccination) and to the characteristics of the target population. In general, measuring the effectiveness of CT, testing and isolation interventions is a complex task, being the type of evidence required hard to obtain (i.e., randomization of interventions is not ethically acceptable); hence, available evidence is mainly based on modelling techniques or on proxy data [13]. With regards to the target population, most people were not officially registered with the Regional Health Service and/or included in the census of resident population, hampering the possibility to monitor the impact of health interventions on their health status. The ECDC specifically pointed out at the importance of collecting and disseminating data on COVID-19 outcomes, testing and vaccination uptake in migrant populations [2]. Greater efforts are needed to ensure the identification of process and outcome indicators in the early phases of interventions, also promoting a stronger data culture into health services.

In conclusion, the COVID-19 pandemic highlighted the need for health systems to respond to sudden shocks, adapting and reorganizing the provision of services in a flexible way [14, 15]. The response to the health needs of migrant and vulnerable populations has long been confronted with this demand for flexibility, being the traditional offer of public health programmes and services often ineffective to reach these groups. Our intervention strategy, based on a strong collaboration between different actors, on a deep context analysis, and on the active involvement of beneficiaries, provides useful elements for the development of public health interventions not limited to the sole management of the COVID-19 emergency, or other epidemic events. The awareness of health professionals on this type of approach, taking into account the complexity of health needs and health systems, will be paramount to encourage the development of innovative interventions. At the same time, it will be necessary to promote data collection and the systematization of available experiences and evidence on intervention models addressed to migrants and HTR groups, in order to support the planning of future initiatives.
